# Lepidopteran Caterpillars in Ambers from Lebanon, Myanmar, Mexico, and the Dominican Republic Indicate Diversification of Defensive Structures and Increasing Abundance over Time

**DOI:** 10.3390/insects17100991

**Published:** 2026-09-25

**Authors:** Joachim T. Haug, Joshua Gauweiler, Fenja I. Haug, Gideon T. Haug, Viktor Baranov, Zaw Dan, Carsten Gröhn, Damien Le Brizoual, Patrick Müller, Vincent Perrichot, Yinan Wang, Marie K. Hörnig, Carolin Haug

**Affiliations:** 1 Biocenter, Ludwig-Maximilians-Universität München, Großhaderner Str. 2, 82152 Planegg-Martinsried, Germany; joachim.haug@palaeo-evo-devo.info (J.T.H.);; 2 GeoBio-Center at LMU, Richard-Wagner-Str. 10, 80333 München, Germany; 3 Cytology and Evolutionary Biology, Zoological Institute and Museum, University of Greifswald, Soldmannstr. 23, 17489 Greifswald, Germany; 4 Fakultät für Biowissenschaften, Universität Heidelberg, Im Neuenheimer Feld 234, 69120 Heidelberg, Germany; 5 Estación Biológica de Doñana-CSIC, Consejo Superior de Investigaciones Científicas, Avd. Americo Vespucio 26, 41092 Sevilla, Spain; 6 Kachin Amber Research Institute, 4-11/#2, 01061 GHW9+336 Sumprabum, Kachin State, Myanmar; uzd@kachinamber.com; 7 Independent Researcher, 21509 Glinde, Germany; 8 Géosciences Rennes, Centre National de la Recherche Scientifique (CNRS), Univ Rennes, UMR 6118, Campus de Beaulieu bat. 15, 263 Avenue du Général Leclerc, 35000 Rennes, Francevincent.perrichot@univ-rennes.fr (V.P.); 9 Independent Researcher, 66482 Zweibrücken, Germany; 10 Independent Researcher, Arlington, VA 22202, USA; 11 Medical Biology and Electron Microscopy Center, University Medical Center Rostock, Strempelstr. 14, 18057 Rostock, Germany; marie.hoernig@palaeo-evo-devo.info

**Keywords:** Kachin amber, Cretaceous, Miocene, Lepidoptera, Neuroptera

## Abstract

**Simple Summary:**

Caterpillar fossils are rare; the known ones are mostly preserved in amber. In this study, we present twelve additional caterpillar fossils in four different types of ambers plus one caterpillar in Tanzanian Holocene copal. For most of these it proved difficult to find out about their identity (i.e., which species or higher group they belong to). Despite this challenge we measured the length of their defensive structures, hairs (setae), and spines, and compared these between the types of amber and according to age. We furthermore compared how many caterpillars were found in ambers in relation to larvae of another extensively sampled group in amber, namely Neuroptera (lacewings). Our results show that the maximum length of hairs or spines becomes successively longer over time, reaching the highest extreme in the modern fauna. Yet, already in the caterpillars in about 20 million years old Dominican amber very long setae were present. Over time, increasingly more caterpillars in relation to lacewing larvae are found in resins. Combining both aspects indicates an increase in diversity of morphological structures of caterpillars and an increase in abundance. At the same time lacewing larvae lost diversity and abundance towards the modern fauna. The results demonstrate that fossils, for which the species or higher group can not be determined, also carry ecologically meaningful information and should not be ignored.

**Abstract:**

Caterpillars are still rare as fossils, but most commonly preserved in ambers. We report here twelve additional specimens from four different types of ambers plus one caterpillar in Tanzanian Holocene copal. Most of these prove to be challenging to be dealt with from a taxonomic point of view. Despite this challenge we measured protruding defensive structures, setae and spines, and compared these between the types of amber and according to age. We furthermore compared the relative abundance of caterpillars in ambers in relation to larvae of another extensively sampled group in amber, namely Neuroptera. Our results show that the maximum length of protruding structures becomes successively longer over time, reaching the highest extreme in the modern fauna. Yet, already in the Miocene very long setae have occurred, as demonstrated by a new fossil from Dominican amber. Relative abundances of caterpillars in relation to lacewing larvae in resins increase significantly and steadily over time. Combining both aspects indicates an increase in diversity of morphological structures of caterpillars and an increase in abundance. At the same time lacewing larvae lost diversity and abundance towards the modern fauna. The results demonstrate that taxonomically challenging fossils carry ecologically meaningful information and should not be ignored.

## 1. Introduction

Lepidopteran caterpillars are ubiquitous elements of the modern terrestrial fauna, occurring in rather large abundances in some instances [1]. As the group of butterflies and (other) moths is also very species-rich [2,3,4], lepidopteran caterpillars represent an enormous biomass. With their major ecological role of feeding and growing, they transform large masses of plant material into protein-rich body mass [5], therefore representing an attractive food source for many other animals, most notably birds [6,7]. Therefore, not only do adult lepidopterans represent important components of our ecosystems as pollinators, but their larvae are also crucial components in our modern food webs.

We should assume that this has been similar in past ecosystems, at least back into the Mesozoic. Still, overall fossils of lepidopterans are rare [8,9], including their larvae. While there are some examples of mineralized fossils in sedimentary rocks [10,11,12,13], most lepidopteran caterpillar fossils have been found in fossil resins, hence amber. So far there are about a dozen larvae known from Cretaceous ambers [14,15,16,17,18,19,20], about ten times this number in Eocene ambers [15,21,22,23,24,25,26,27,28,29,30,31,32,33,34,35,36,37,38,39,40,41], and 20 larvae in Miocene (or Miocene plus Oligocene) ambers [15,25,42,43,44,45,46,47,48,49]. In addition, very few specimens have been reported from non-fossil resins [50].

This overall low number of larvae is partly surprising when compared to larvae of other groups which are much less species-rich, such as Neuroptera or Raphidioptera, but are known from a quite astonishing number of larval specimens in amber [5,51,52,53,54,55,56,57,58,59,60,61,62,63,64,65,66,67,68,69,70,71,72,73,74,75,76,77,78,79,80,81,82,83,84,85]. Other groups which are closer to the species richness in Lepidoptera such as Coleoptera [86,87,88,89,90,91,92,93,94,95,96,97,98,99,100] or Diptera [101,102,103,104,105,106] are also known from quite a large number of fossil larval specimens preserved in resins. It remains unclear whether the rarity of lepidopteran caterpillars in amber is caused by true rarity in the original fauna, or if it is caused by a preservational bias, or if it is the result of a biased report.

Here we report new fossil specimens of lepidopteran caterpillars from four different types of ambers: Lebanon, Myanmar, Mexico, and the Dominican Republic. Additionally, we report one lepidopteran caterpillar in Tanzanian Holocene copal, hence non-fossil resin. We compare their relative abundance between the types of amber and according to age in order to better understand the seeming rarity of their record.

## 2. Material and Methods

### 2.1. Material

We report here twelve new caterpillars preserved in eleven pieces of amber and one lepidopteran caterpillar preserved in a piece of Tanzanian Holocene copal.

The caterpillar preserved in Tanzanian Holocene copal has the repository number IGR.TZ-287 (housed in the Geology Department and Museum of the University of Rennes, France). About 9 kg of this copal, including this specimen, was collected from Jangwani Beach, Dar-es-Salaam, by a local resident. Then, 14C analysis performed on six pieces from this sample indicated an age between 185 ± 30 and 2655 ± 30 years BP.

Six pieces of amber with seven of the new caterpillars are of Miocene age. Four pieces are from the Dominican Republic: SMNS Do 359-K (Staatliches Museum für Naturkunde Stuttgart), PED 1524, PED 1585, and PED 2015 (the latter with two specimens, PED 2015a and PED 2015b; Palaeo-Evo-Devo Research Group Collection of Arthropods, Ludwig-Maximilians-Universität München, Germany). The other two are from Mexico: MEX 027 (part of the collection of one of the authors, P.M.) and PED 2032.

Five specimens are preserved in Cretaceous ambers. Four pieces are Kachin amber, Myanmar: cn-05-25 (Kachin Amber Research Institute), BUB 3968 (part of the collection of one of the authors, P.M.), PED 2352, and PED 4046. One of these is from Lebanon: SMNS LB-250-2.

For comparison, we also present a new lacewing larva from Miocene Dominican amber. It is part of the collection of one of the authors (C.G.) and has the repository number CCGG 10059.

PED 1524, 2015, 2352, and 4046 were legally purchased on the trading platform ebay.com from various traders (terratreasures, amber_resin_fossils, and burmite-miner). PED 1585 was purchased from the trader Ambra Greco.

### 2.2. Documentation Methods

Most specimens were documented on a Keyence VHX-6000 digital microscope (Keyence, Osaka, Japan). All images are compound images combining images from different focal planes (*z*-axis) and adjacent image details (*x*/*y*-axes). In addition, the HDR function of the microscope was used.

Specimen cn-05-25 was documented with a macrophotographic setup, using a Fujifilm X-E5 camera (Fujifilm, Tokyo, Japan) with a Laowa 65mm f/2.8 2× Ultra Macro APO objective (Venus Optics, Hefei, China). For the transmitted light we used an LED VIJIM 50 W light panel (VIJIM, Shenzhen, China). An image stack was created with approximately 12–18 µm steps with the aid of a focus stacking rail.

Micro-computed tomography (µCT) of PED 1524 was performed using a Zeiss Xradia MicroXCT-200 (Carl Zeiss Microscopy GmbH, Jena, Germany) with the following settings: 4× objective, 40 kV, 8 W, 1.5 s exposure time, and binning 2. The resulting projections were processed with XMReconstructor software (version 8.1, Carl Zeiss Microscopy GmbH, Jena, Germany) with binning 1 resulting in tiff-image stacks, with a system-based calculated voxel size of 4.17 µm, and image size of 1014 × 1014 px. Visualizations of the µCT data were obtained with Drishti 2.4 [107].

### 2.3. Relative Abundances

Absolute numbers are challenging to interpret in fossils; we therefore compare relative ones. As in Haug et al. [48], we used lacewing larvae as a comparison as such larvae in ambers have been quite thoroughly explored. We used all specimens found in the literature, reporting them here and listing them in our collection database (including some yet-to-be-published lacewing specimens). We want to emphasize that we use the books of Yahya [31,32,46,57] exclusively as image source, but completely reject his creationist ideas.

### 2.4. Measurements

For a quantitative comparison we measured the relative length of protruding structures, setae and spines. As the literature often does not provide scales for measuring absolute length, we used a ratio: the maximum length of the protruding structure in relation to the body diameter. Concerning the defensive capacities, this ratio is expected to be rather informative as it simply provides an estimate of the gain in physical barrier against a predator. For smaller predators such as ants or beetles protruding hairs and spines can keep the predator away, so that they cannot reach the body. For predators such as birds or lizards, the protruding structures may make them large enough so that they cannot be swallowed.

The longest protruding structure was always measured. In most cases it proved difficult to clearly differentiate spines from setae (setae have a distinct joint, whilst spines lack this), or, if both structures were present, to clearly see the distal end of the spines (as they would often be concealed by setae). Therefore, the sub-sample for spines is much lower. As many images in the literature are not standardized, we sometimes had to measure the trunk diameter in dorsal view, sometimes in lateral view, and sometimes in oblique view. This approach adds noise to the dataset, but should still provide us a rough estimate; it also fits well with the preservation condition of the fossils, in which the direction of view is also not standardized. In some cases the caterpillars bear so many setae that the trunk could not be measured with confidence; in such cases we did not include the specimen in the dataset. We used the atlas of Nippe [108] as a basis for the modern fauna.

In addition, we compared the change in the seta length over ontogeny. We found data for four growth series of extant species with apparent setae. We plotted the ratios of the maximum length of the protruding structure in relation to the body diameter over the stages to see trends in relative length in post-embryonic development.

## 3. Results

### 3.1. Lepidopteran Caterpillar in Holocene Copal

Specimen IGR.TZ-287 is preserved in Tanzanian Holocene Copal. The body is elongate about 2.3 mm long. The head capsule is visible, but mouthparts are not discernible. Thorax legs are preserved on all thorax segments (Figure 1a,b). They are conical, tapering distally, and appear well sclerotized. Appendages are apparent on abdomen segments 3–6 and trunk end (prolegs, leglets; Figure 1b). Appendages on abdomen segments 3–6 bear crochets, with several hooks distally (Figure 1b). The body surface has many folds and setae longer than thorax legs.

### 3.2. Lepidopteran Caterpillars in Miocene Ambers

Lepidopteran caterpillars from Miocene ambers were summarized in 2022 [48]. At that time, there were 19 occurrences in total from Mexican and Dominican ambers. Solis et al. [49] reported another caterpillar from Dominican amber, bringing the sum up to 20. Additional new specimens are listed below:

(1) Specimen PED 1524 (specimen 21) is preserved in Dominican amber. The specimen is accessible in the dorsal (Figure 2) and ventral view (Figure 3 and Figure 4) and has also been scanned in a µCT, making it accessible in several directions (Figure 5). The body is organized into the head and elongated trunk region, with the body in total presumably having 20 segments (yet not all segments are apparent; Figure 2a,c, Figure 3, Figure 4a,b, and Figure 5a–c).

The head forms a distinct globular capsule, slightly wider than it is long (Figure 2b, Figure 3b, Figure 4c,d and Figure 5d). There are multiple long setae on the head capsule, with the exact arrangement (chaetotaxy) not discernible. Multiple distinct structures are apparent on the head (Figure 4c,d and Figure 5d–f), including multiple stemmata (Figure 4c,d). The ocular segment is recognizable by its appendage derivatives, labrum, and well distinguishable (Figure 4c,d and Figure 5e,f). The appendage of post-ocular segment 1, the antenna, is short (Figure 4c,d and Figure 5d–f). Post-ocular segment 2 (intercalary segment) does not have externally visible structures. Post-ocular segment 3 has a distinct mandible, but the details are not accessible (Figure 4c,d and Figure 5d–f). The appendage of post-ocular segment 4, the maxilla medio-proxially bears an endite (galea?); distally it has an elongate palp, which is subdivided into four elements (Figure 4c,d and Figure 5d–f). Post-ocular segment 5 has appendage derivatives, labium, which distally bears a pair of small palps (Figure 4c,d and Figure 5d–f).

The trunk has twelve more or less well-apparent units (Figure 3, Figure 4a,b and Figure 5a–c); the anterior eleven appear to be true segments. Each trunk segment has at least two pairs of verrucae (elevated sclerites), each with multiple long setae (Figure 2c). The anterior three trunk segments (post-ocular segments 6–8; pro-, meso-, and metathorax) ventrally have paired sclerotized appendages (thorax legs), subdivided into several elements (Figure 4a,b). All thorax legs have setae arranged in a ring around some elements (Figure 4e).

Further posterior segments (abdomen) gradually become longer but not wider (Figure 2a,c, Figure 3, Figure 4a,b, and Figure 5c). All segments have two paired verrucae with long setae. The first two abdomen segments do not have appendages (post-ocular segments 9 and 10). Abdomen segments 3–6 (post-ocular segments 11–14) each have two paired appendages (prolegs, leglets). Appendages are not subdivided, distally with crochets (at least seven; Figure 4e). Abdomen segments 7 and 8 (post-ocular segments 15 and 16) do not have appendages (Figure 4a,b).

The trunk end (most likely compound structure of several, not distinguishable post-ocular segments 17–19) has a distinct pair of appendages (prolegs, leglets). Appendages have crochets and multiple long setae all over the appendage (Figure 4a,b).

(2) Specimen PED 1585 (specimen 22) is preserved in Dominican amber. The specimen is accessible in oblique dorsal (Figure 6a,b) and ventral view (Figure 6c). The body is organized into a head and elongated trunk region, with the body in total presumably having 20 segments (Figure 6). Overall, the body appears to be rather flat.

The head forms a distinct globular capsule, wider than it is long, with few rather short setae, the exact arrangement (chaetotaxy) not being reconstructable. No details of structures on the head are apparent due to the small size of the specimen (Figure 6c).

The trunk has twelve more or less well-apparent units, and the anterior eleven appear to be true trunk segments. Trunk segments have numerous setae, with the setae being longer than those on the head. The anterior three trunk segments (post-ocular segments 6–8; pro-, meso-, and metathorax) are shorter than they are wide, about as wide as the head capsule; the first segment (prothorax) is slightly longer than the other two. The first segment has two structures that presumably represent appendages (thorax legs), but the appendage length or subdivision is not well apparent (Figure 6c). The second segment has one visible structure that presumably represents an appendage (likely a pair of thorax legs). The third segment does not have visible appendages, but probably also has a pair of appendages.

All further posterior segments (abdomen) are similar to the anterior ones; shorter than they are wide, but slightly thinner than further anterior segments (Figure 6a,b). Abdomen segments 3–6 (post-ocular segments 11–14) have small paired structures that likely represent short appendages (prolegs, leglets; Figure 6c).

The last trunk unit (trunk end) is most likely a compound structure of several, not distinguishable segments (post-ocular segments 17–19?), with a distinct pair of appendages (prolegs, leglets; Figure 6a,b). No details are accessible.

(3) Specimen SMNS Do 359-K (specimen 23) is preserved in Dominican amber. The specimen is observable in ventral (Figure 7a,b) and dorsal (Figure 7c) view. The specimen is oblique in the amber, with one region being deeper embedded from each side. Therefore, there is slightly better access to posterior details in ventral view and anterior details in dorsal view. With the aid of glycerol it was possible to obtain a slightly less distorted dorsal view (Figure 7d). The body is organized into a head and elongated trunk region; the body in total presumably has 20 segments.

The head forms a distinct globular capsule, slightly longer than it is wide, with few short setae; the exact arrangement (chaetotaxy) is not reconstructable. No details of structures on the head are apparent, except for an outline of the labium (Figure 7a,b).

The trunk has twelve more or less well-apparent units; the anterior eleven appear to be true trunk segments. The trunk segments have few short setae, with the setae being similar in length to those on the head. The anterior three trunk segments (post-ocular segments 6–8; pro-, meso-, and metathorax) are longer than the other segments, and wider than the head capsule. The first segment (prothorax) is slightly shorter than the other two. The dorsal side of the first trunk segment is lightly sclerotized. All three anterior trunk segments have paired structures that represent appendages (thorax legs), but the appendage subdivision is not well apparent (Figure 7a,b). Appendages get progressively longer each segment, with the appendages shortest on the first segment and longest on the third. Overall the appendages are small.

All further posterior segments (abdomen) are similar to the anterior ones; all segments are much shorter than they are wide. There are few short setae on the abdomen segments. The first two abdomen segments (post-ocular segments 9 and 10) do not have appendages. Abdomen segments 3–6 (post-ocular segments 11–14) each have a pair of small structures that likely represent appendages (prolegs, leglets). Appendages do not appear subdivided. Abdomen segments 7 and 8 (post-ocular segments 15 and 16) do not have apparent appendages. The trunk end is most likely a compound structure of several, not distinguishable segments (post-ocular segments 17–19?), with a distinct pair of appendages (prolegs, leglets). No details are accessible.

(4) Specimen PED 2015a (specimen 24) is preserved in Dominican amber. It is ventrally folded, and obliquely laterally accessible (Figure 8a,b,d). Details of the head are largely obscured (Figure 8a). Appendages of thorax segments (thorax legs) are visible, but details are not accessible due to the optical properties of the amber. Abdomen segments 1–5 are elongate and without appendages. Abdomen segment 6 has prominent appendages (prolegs, leglets; Figure 8c). Abdomen segment 7 does not have appendages. All remaining segments forming the trunk end have a prominent pair of appendages (prolegs, leglets). The body surface has numerous folds and wart-like seta sockets (Figure 8a).
(5) Specimen PED 2015b (specimen 25) is preserved in Dominican amber. It is in the same amber piece as specimen 24. The specimen is smaller than specimen 24 and incomplete; the posterior end is missing (Figure 8f,g). The specimen is accessible in oblique antero-lateral (Figure 8e) and postero-lateral (Figure 8f) view. The head has mouthparts, without details. Appendages of the thorax segments are apparent (thorax legs), and subdivisions are discernible. Further details are not accessible. Abdomen segments 1–5 are elongate and without appendages. Abdomen segment 6 is incomplete, with one prominent appendage preserved (prolegs, leglets; Figure 8f,g). All further posterior structures are missing, outside of the amber piece.
(6) Specimen PED 2032 (specimen 26) is preserved in Mexican amber. The specimen appears overall complete and accessible in oblique dorsal view (Figure 9a,b); however, it seems to have undergone shrinkage, which obscures many details. In oblique ventral view additional disturbances in the amber limit the accessibility (Figure 9c). The head is apparent with possible antenna discernible; mouthparts are not accessible. Thorax appendages (thorax legs) are apparent on the prothorax and metathorax. Abdomen appendages (prolegs, leglets) are visible on abdomen segments 3–5 and at the posterior end of the trunk; it is unclear whether abdomen segment 6 lacks an appendage or whether it is not accessible.
(7) Specimen MEX 027 (specimen 27) is preserved in Mexican amber. It appears to be overall complete, yet the anterior part is very close to the rim of the amber piece, limiting the accessibility. Additionally the amber piece has a lot of disturbances, further concealing some details. The head is apparent, close to the rim of the amber piece, with possible antennae protruding from it (Figure 10c). No details of the mouthparts are accessible. Thorax appendages (thorax legs) are vaguely visible, with the exact segment identity being unclear (Figure 10c). Stout appendages (prolegs, leglets) on abdomen segment 3–6 are apparent (Figure 10a,b), with more elongate appendages on the trunk end (Figure 10d). The body surface has numerous folds and few short setae.

### 3.3. Lepidopteran Caterpillars in Cretaceous Ambers

The record of lepidopteran caterpillars in Cretaceous ambers, including ambers from Canada, Lebanon, and Myanmar, has been summarized in Haug and Haug [18] and updated in Gauweiler et al. [19], summing up to ten clear cases. An additional specimen since then has been reported in Haug et al. [20] (figure 7c, p. 10), bringing the number up to eleven. Additional new specimens are listed below:

(1) Specimen BUB 3968 (specimen 12) is preserved in Kachin amber, Myanmar. The specimen is accessible in ventral view (Figure 11a,b) and is well preserved. Only the optical properties of the amber limit access to certain aspects. The body is overall very elongate. The head capsule has short antennae, and some mouthparts (maxillae, labium) are discernible, but without clear details (Figure 11c). Thorax legs are preserved on all segments and are relatively short compared to the segment diameter (Figure 11d). Appendages are also apparent on abdomen segments 3–6 (prolegs, leglets; Figure 11e) and the trunk end (Figure 11a,b). The body surface has setae longer than the thorax legs.
(2) Specimen PED 2352 (specimen 13) is preserved in Kachin amber, Myanmar. The specimen has a very elongate body. The head capsule has short antennae, not protruding beyond the rim of the head capsule. Some mouthparts (maxillae, labium) are discernible, but without clear details (Figure 12c). Thorax legs are preserved on all thorax segments. They are relatively short compared to the segment diameter (Figure 12d), conical, strongly tapering distally, and well sclerotized, bearing some longer setae. Appendages are also apparent on abdomen segments 4, 6, and the trunk end (prolegs, leglets; Figure 12a–c). Appendages on abdomen segment 4 have crochets preserved (Figure 12d). They are not fully accessible, but we can estimate 18 hooks on each appendage (Figure 12d). The body surface has many folds and setae longer than thorax legs. Sockets of setae appear darker and wart-like.
(3) Specimen PED 4046 (specimen 14) is preserved in Kachin amber, Myanmar. The specimen has a very elongate body. The head capsule has short antennae, with some mouthparts (maxillae?, labium?) discernible, but without clear details (Figure 13a,b). Thorax legs are preserved on all thorax segments (Figure 13a,b). They are conical, tapering distally, and well sclerotized. Appendages are also apparent on abdomen segments 3–6 and the trunk end (prolegs, leglets; Figure 13a,b). Appendages on abdomen segments 4–6 have crochets preserved (Figure 13c). They are not fully accessible, but we can estimate 22 hooks on each appendage (Figure 13c). The body surface has many folds and setae not longer than the thorax legs.
(4) Specimen cn-05-25 (specimen 15) is preserved in Kachin amber, Myanmar. The specimen has a rather large elongate body of about 10 mm in length (Figure 14a,b). The head capsule has some mouthparts (maxillae?, labium?) discernible, but without clear details (Figure 14c). Thorax legs are preserved on all thorax segments (Figure 14a,b). They appear to be conical and tapering distally. Appendages are also apparent on abdomen segments 3, 5, and 6 (unclear for abdomen segment 4) as well as the trunk end (prolegs, leglets; Figure 14d,e). The body surface has few setae visible.
(5) Specimen SMNS LB-250-2 (specimen 16) is preserved in Lebanon amber. The specimen is highly incomplete, and only the posterior part of the trunk is preserved (Figure 15a,b). The supposed abdomen segments 4–6 have stout appendages (prolegs, leglets). The trunk end also has a pair of short conical appendages (Figure 15a,b).

### 3.4. Relative Abundances

For Miocene ambers we now have 27 reported caterpillars. In addition to the already-known 16 lacewing larvae from that time period [109], we report here another one from Dominican amber (CCGG 10059, Figure 16), hence summing up to 17 Miocene lacewing larvae. These numbers result in a ratio of caterpillars to lacewing larvae of about 1.59 (Figure 17a). For the Eocene we so far have only an estimate for the lepidopteran caterpillars; focused collection efforts by some enthusiasts [29,30,34] bring the number up to about 120, although it needs to be mentioned that many of them are potentially empty cases. The lacewing larvae in Eocene ambers are dominated by the group Nevrorthidae, for which several amber pieces are known to have specimens occurring together in a single piece of amber, sometimes more than 30 [59,84], making a total of 115 lacewing larvae. With this, the ratio of caterpillars to lacewing larvae for the Eocene is 1.04. For the Cretaceous we now have 16 lepidopteran caterpillars. For the lacewing larvae the situation is very different: combining the literature with our collection database leads to a sum of about 900 lacewing larvae in the Cretaceous, providing a ratio of caterpillars to lacewing larvae of 0.02.

### 3.5. Relative Length of Protruding Structures

Measuring setae and spines (measurements in Supplementary Table S1) and plotting them over time reveal a rather clear pattern. The maximum lengths of setae and spines increased over time, being shortest in the Cretaceous and becoming progressively longer in Eocene and Miocene, reaching their greatest length in the modern fauna (Figure 17b). Only the inclusion of specimens in non-fossil resins breaks the general pattern partly (Figure 17b), but they have only been separated for highlighting and are basically part of the modern fauna.

For changes over ontogeny, we can recognize that in at least one species the setae seem to become longer (Figure 17c). In other species, they become shorter during ontogeny, with the stage 1 larvae having the longest setae (Figure 17c).

## 4. Discussion

### 4.1. Identity of the Specimens

All specimens are identified as lepidopteran caterpillars based on their overall appearance and especially the arrangement of appendages on the abdomen. Further-reaching interpretations remains challenging at least for some of the specimens.

Specimen IGR.TZ-287 in Holocene copal resembles the two caterpillars described in Gauweiler et al. [50] to a certain degree. The latter two specimens were tentatively interpreted as representatives of Erebidae (Noctuoidea), mainly based on the arrangements of spine-bearing humps occurring only in the two ingroups Arctiinae and Lymantriinae [50]. Further identification was not possible due to a lack of accessible characters, which is also the case in IGR.TZ-287. Therefore, we interpret IGR.TZ-287 as a possible larva of a species of Erebidae. Representatives of Erebidae, especially of Arctiinae and Lymantriinae, occur in Tanzania today [115,116,117], which supports our interpretation.

For PED 1524 the appendage arrangement, long setae, and elongated verrucae on the first trunk segment suggest the caterpillar is a representative of Lymantriinae. Caterpillars with such long setae are very rare in the fossil record [50], and the new specimens exhibit the relatively longest setae known so far; the specimen therefore represents an important new find.

Specimen PED 1585 is very small and therefore many details are not visible. The overall morphology is not compatible with an interpretation as a representative of, for example, Geometridae (due to the arrangement of the prolegs) or Lymantriinae (due to the absence of long setae or spines), but clear positive identifiers for any other group are also not accessible.

For SMNS Do 359-K, the appendage arrangement, sparse setae, and apparently slightly sclerotized dorsal side of the first trunk segment identify this caterpillar as a possible representative of Tortricidae [118]. Unfortunately, many details are not accessible as the amber is not very clear and causes optical distortions; therefore, a more-detailed identification is not easily possible.

For the two specimens in amber piece PED 2015, i.e., the larger PED 2015a and the smaller PED 2015b, the arrangement of the posterior end, with a very prominent appendage on abdomen segment 6 positioned close to the trunk end, clearly identifies both as representatives of Geometridae. This is only the second case of finding two caterpillars together in a single amber piece. The other finding includes two specimens in a piece of Baltic amber ([30] figure 264 p. 65). It is also the first documented case of two different growth stages preserved together, the first such case for Geometridae, and the first record of two caterpillars in a single piece of Dominican amber.

For specimen PED 2032, the situation is somewhat similar to that of PED 1585, as few diagnostic details are accessible. It remains unclear if an appendage is present on abdomen segment 6. If it is absent, this would be a signal in the direction of the group Gracilariidae, yet as the point remains unclear, this also accounts for the further identification of this specimen.

Specimen MEX 027 likewise lacks prominent identifying characters. Additionally, many details like mouthparts or crochets are simply not accessible.

The four specimens from Kachin amber, BUB 3968, PED 2352, PED 4046, and cn-05-25, all share certain characters, although the appendages are slightly more elongate in PED 4046. Compared to other finds in Cretaceous ambers [19], these are much more “normal” in overall appearance. Many caterpillars in Cretaceous amber lacked the appendage on abdomen segment 6 (exceptions in [19]), and so far none of them preserved the crochets. The new finds are therefore adding a much more “normal” appearance to the diversity of caterpillars in the Cretaceous. However, they also lack prominent characteristics for further narrowing down their exact relationships.

Specimen SMNS LB-250-2 is very incomplete, but still an important find. A previous find of a caterpillar in Lebanon amber [15] is so far the oldest report of a lepidopteran caterpillar, and it represents a leaf-mining form. Although incomplete, the present specimen constitutes only the second caterpillar known from the same time interval and is therefore already significant. The abdomen appendages are rather stout but stronger expressed than in the known leaf-mining form from Lebanon amber; however, it cannot be excluded that the appendages were still sufficiently short to allow a leaf-mining lifestyle. A factor arguing against such a lifestyle is the rather round-appearing diameter of the specimen. Yet, also here we are left with uncertainties due to the rather incomplete preservation of the specimen.

As is apparent, it is very challenging to further identify most of these fossil larvae. This difficulty seems a main reason as for why many fossil larvae are simply not reported. It has been suggested that fossil larvae should only be treated by taxonomic specialists [119], yet the difficulties seem to decrease interest in doing exactly that. Larvae are simply not easy to handle within a strict taxonomic framework, yet quantitative approaches can still yield useful information [120]. Therefore, it is important to report such larvae despite taxonomic uncertainties, in order to avoid introducing further bias into quantitative analyses.

### 4.2. Relative Abundances

Relative abundances of lepidopteran caterpillars versus lacewing larvae were already explored in Haug et al. [48]. At that time the Eocene stood out with the highest relative abundance of lepidopteran caterpillars, while the Miocene had a slightly lower abundance. With the new specimens, and especially more lacewing larvae in the Eocene, the picture has changed, and we see a continuous change in abundance from the Cretaceous to the Miocene with lacewings declining and lepidopterans becoming more common, with the ratios rising from 0.02 (Cretaceous) to 1.04 (Eocene) and 1.59 (Miocene). If we expand that into the non-fossil resins, numbers are too low to be reliable, but so far there are four clear cases of caterpillars: the one newly described here from Tanzanian Holocene copal, one from Madagascar Holocene copal [121] (unnumbered figure on p. 191), and two specimens in Gauweiler et al. [50], as well as one of a lacewing [30] (figure 206 p. 55), which would provide a ratio of 4.0. Looking into the modern fauna, caterpillars are much more regularly encountered when walking around than lacewing larvae are, providing the impression of an overall continuous change, though this impression has to be taken with care.

When differentiating the different types of amber available for the Cretaceous, we recognize a difference in Lebanon amber, which is about 30 million years older than the other Cretaceous ambers. Here the ratio is 0.2, hence not as extremely shifted towards lacewings as in the younger Cretaceous ambers. However, the specimen numbers for Lebanon amber are still very low (two caterpillars, ten lacewing larvae [15,64,66]), and therefore unlikely to provide a good measure (although not as poor as for the non-fossil resins). Overall, the general signal remains unchanged: in the Cretaceous ambers, lacewing larvae are much more numerous than caterpillars, compared to younger ambers.

The main limitation of using relative abundances is that the exact underlying causes can be difficult to determine. Changes may result from (1) one group diversifying while another remains stable, (2) one group declining while another remains stable, or (3) one group diversifying while another declines. Therefore, additional measures should be considered to interpret such patterns accurately.

### 4.3. Relative Length of Protruding Structures

We can recognize a clear increase in the relative maximum length of the protruding structures on caterpillars over time. Interestingly we can also recognize that the modern fauna not only has the specimens with the longest structures but also those with the shortest ones (Figure 17b). However, this notion may be due to a problematic mismatch in accessible life stages. Amber seems to prefer the preservation of smaller animals, favoring early larval stages. Entomological research on extant animals focusses mostly on late stages, preferably individuals in their last larval stages. In the latter, the body diameter is often larger in relation to the spines and setae, leading to relatively shorter lengths. This effect may also partly affect the two specimens in non-fossil resins, as these have been the largest specimens preserved in resins [50]. However, with the newly reported specimens we now have a very large specimen in Kachin amber (Figure 14), and we also now have a rather small specimen in Holocene copal (Figure 1).

In addition, measuring the relative lengths over post-embryonic ontogeny in extant species did not reveal a general pattern of setae becoming relatively longer over ontogeny (Figure 17c). On the contrary, for several species the earlier stages seem to in fact have relatively longer setae. This could mean that the signal would be even stronger if we had included more early stages for the extant fauna.

Despite this possible bias, we can indeed see that the defensive structures of lepidopteran caterpillars in the Cretaceous were still rather limited in length in comparison to the later faunas. They became more pronounced and sophisticated, as demonstrated by Eocene fossils, reaching almost modern conditions by the Miocene, being only slightly topped by the modern fauna. This result is congruent with the diversification time of lepidopteran groups with long-haired caterpillars, as indicated by recent molecular phylogenetic reconstructions [122].

Another possible bias could be in the way that the specimens were collected. However, this mostly accounts for the modern fauna, for which the collection of specimens was quite different from that of the fossils. Considering only the fossils preserved in amber, which all have been “collected” in a more similar way and with similar size choices, gives the same signal as with the modern fauna being included.

While this result may only represent a weak signal, it shows that lepidopteran caterpillars are today more diverse concerning their anti-predator strategies, as “naked” caterpillars are still around as well as basically protruding structures of all lengths that have been known in the past. Hence, presumably a diversification of morphological structures over time occurred in lepidopterans.

For lacewings we have reliable morphological data demonstrating a loss of diversity in the morphology (and coupled to this, ecology) of their larval forms since the Cretaceous [120]. Together with the abundance data, this argues for a scenario of type 3: lacewings declined and lepidopterans diversified since the Cretaceous.

### 4.4. Rarity of Caterpillars

It remains to be evaluated if the seeming rarity of caterpillars in resins is a real signal or a bias. We have actively searched for caterpillars in Cretaceous and Miocene ambers and non-fossil resins, as demonstrated by recent studies [18,19,48,50] and this contribution. For Eocene ambers some enthusiasts have also put quite some effort into finding caterpillars [29,30,34]. It is therefore unlikely that there are large numbers present that have not been recognized, at least for the Cretaceous; for the Eocene there seem to indeed be several more specimens in different collections (including private ones), and also for the Miocene at least some more finds are to be expected.

Additionally, the combination of the relative abundances in combination with morphological aspects allows us to evaluate the seeming rarity of caterpillars in resins. Lepidopterans have been known since the Triassic [123], and the lineage diversity had already increased by the Mesozoic, leading to quite some morphological diversity in the later Mesozoic [18]. However, it appears that only after the Cretaceous the morphological diversification took off, leading to significantly increased diversity in the Eocene and reaching a rather modern state by the Miocene. Especially considering that the fossil record is always incomplete, it appears likely that the Miocene was not very different from the modern fauna. Taking this together, the rather low record of caterpillars in the Cretaceous could indeed reflect a true original rarity.

## 5. Conclusions

To sum up, the maximum length of protruding structures in lepidopteran caterpillars becomes successively longer over geological time, with the highest length being reached in the modern fauna. Nevertheless, very long setae have already occurred in the Miocene, demonstrated by a new fossil from Dominican amber. There appears to be a significant and steady increase in relative abundances of caterpillars in relation to lacewing larvae in resins over geological time. In total, an increase in the diversity of morphological structures and abundance of caterpillars appears to have occurred, while lacewing larvae lost diversity and abundance through time.

## Figures and Tables

**Figure 1:**
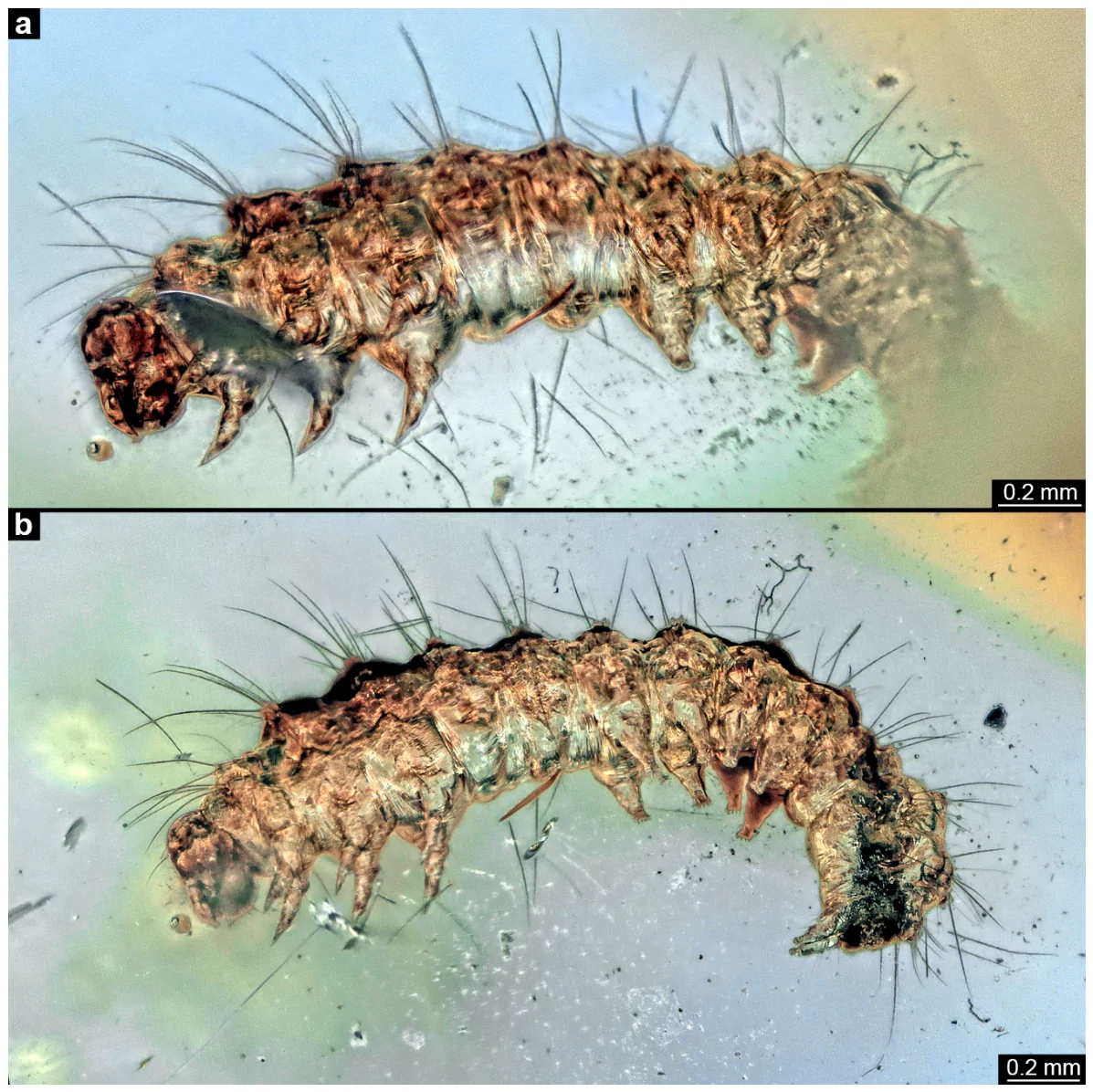
New caterpillar fossil, IGR.TZ-287, from Tanzanian Holocene copal. (**a**) Ventro-lateral view. (**b**) Lateral view at a slightly different angle than in a.

**Figure 2:**
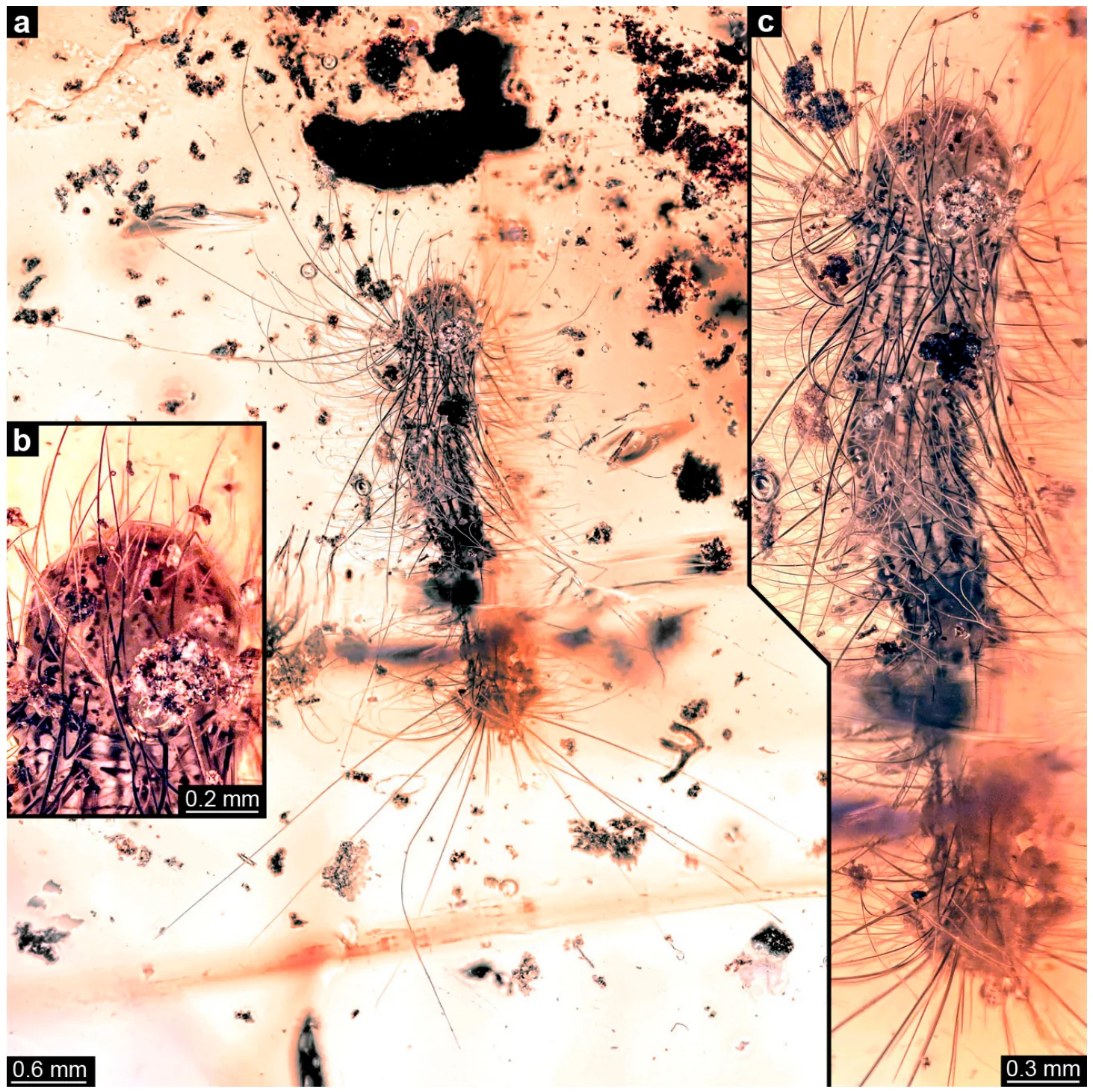
New caterpillar fossil, PED 1524, from Dominican amber in dorsal view. (**a**) Overview of entire specimen; note the long setae. (**b**) Close-up on head. (**c**) Close-up on central body.

**Figure 3:**
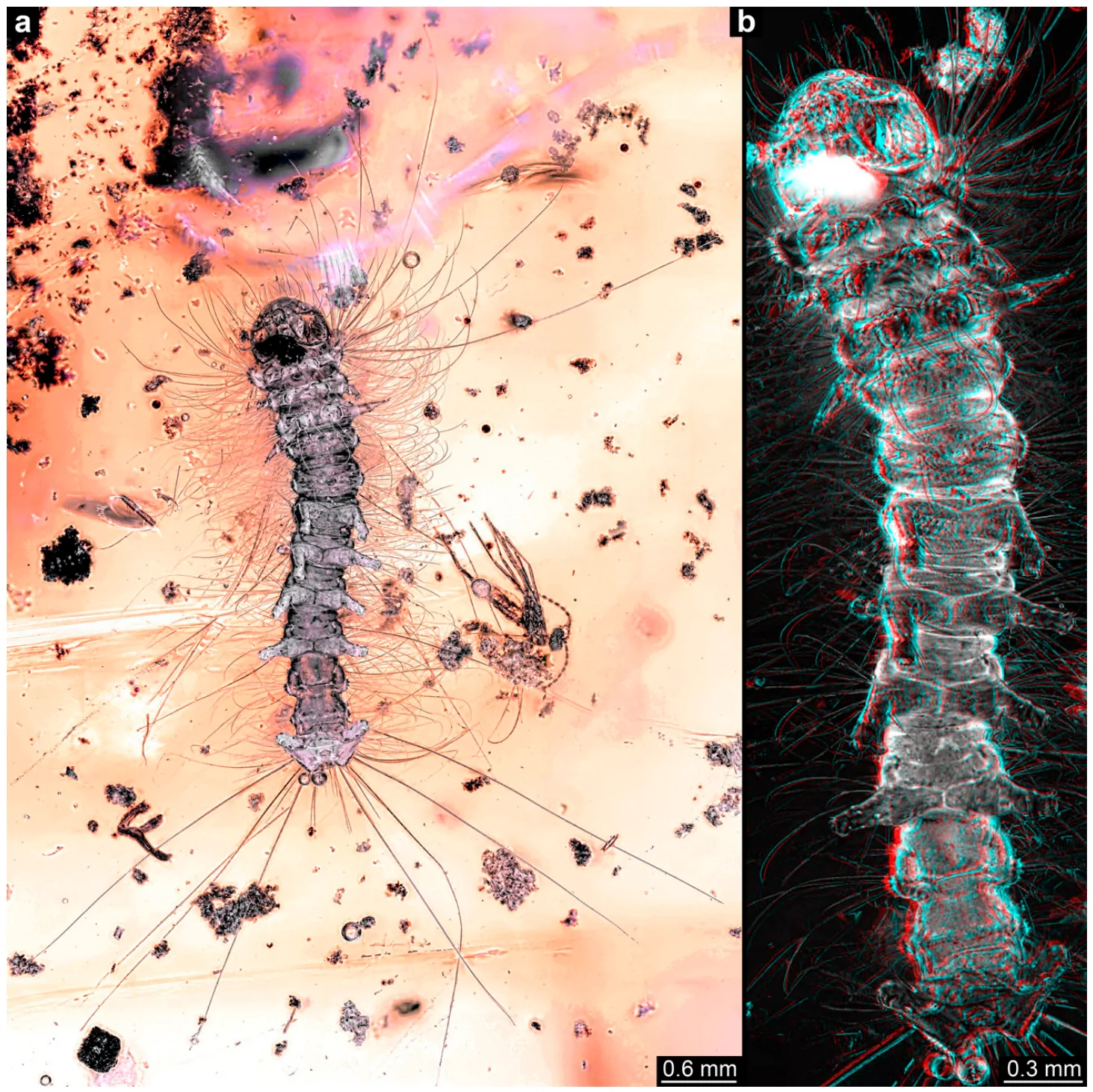
New caterpillar fossil, PED 1524, from Dominican amber in ventral view. (**a**) Overview of entire specimen; note the long setae. (**b**) Close-up on central body as stereo anaglyph; please use red-cyan glasses to view.

**Figure 4:**
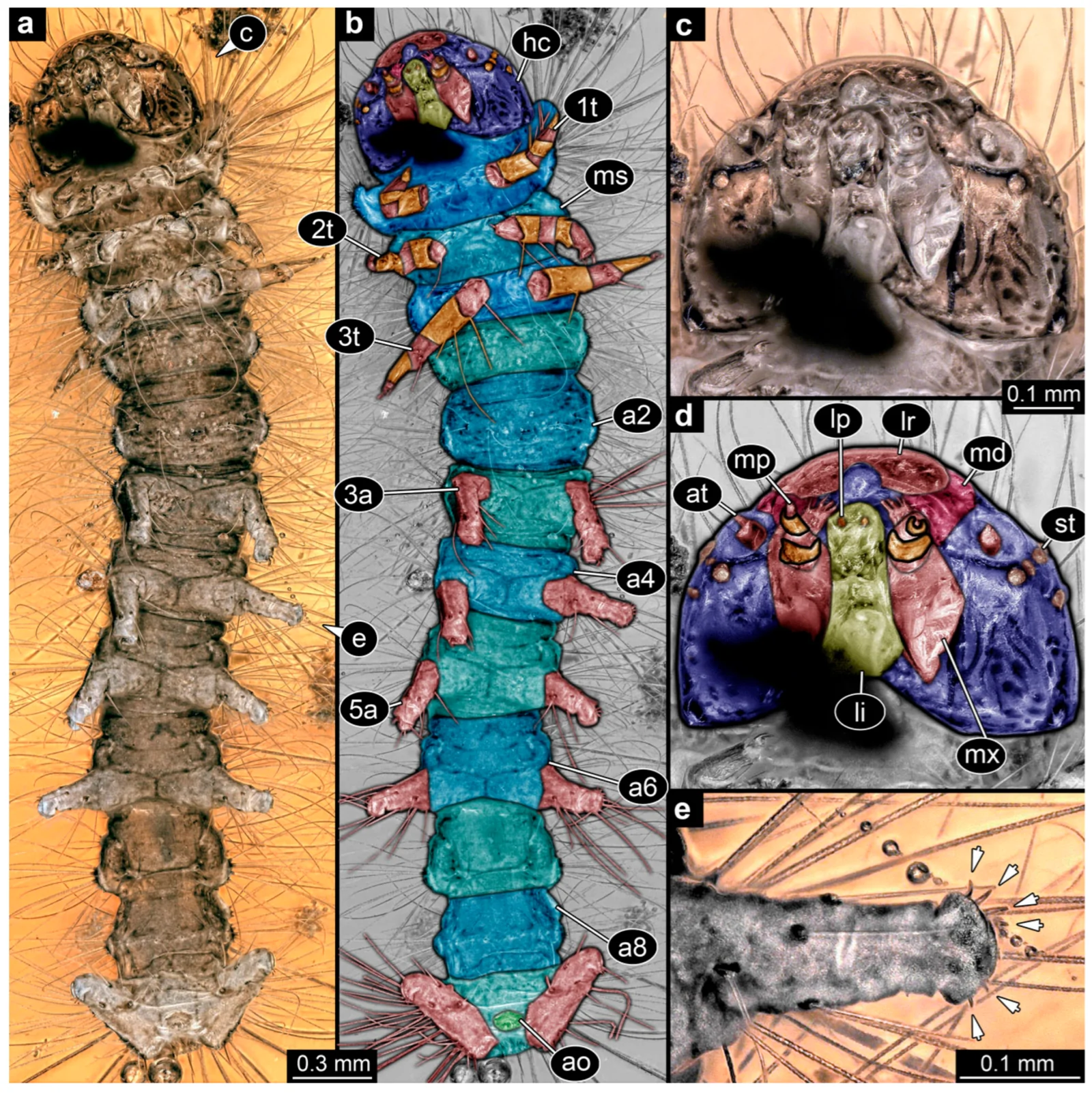
New caterpillar fossil, PED 1524, from Dominican amber in ventral view, continued. (**a**) Overview of central body. (**b**) Color-marked version of (**a**). (**c**) Close-up on head. (**d**) Color-marked version of (**c**). (**e**) Close-up on proleg with crochets (arrows). Abbreviations: 1–3t, appendage of thorax segment 1–3; 3–5a, appendage of abdomen segment 3–5; a2–8, abdomen segment 2–8; ao, anal opening; at, antenna; hc, head capsule; li, labium; lp, palp of labium; lr, labrum; md, mandible; mp, palp of maxilla; ms, mesothorax; mx, maxilla; and st, stemmata.

**Figure 5:**
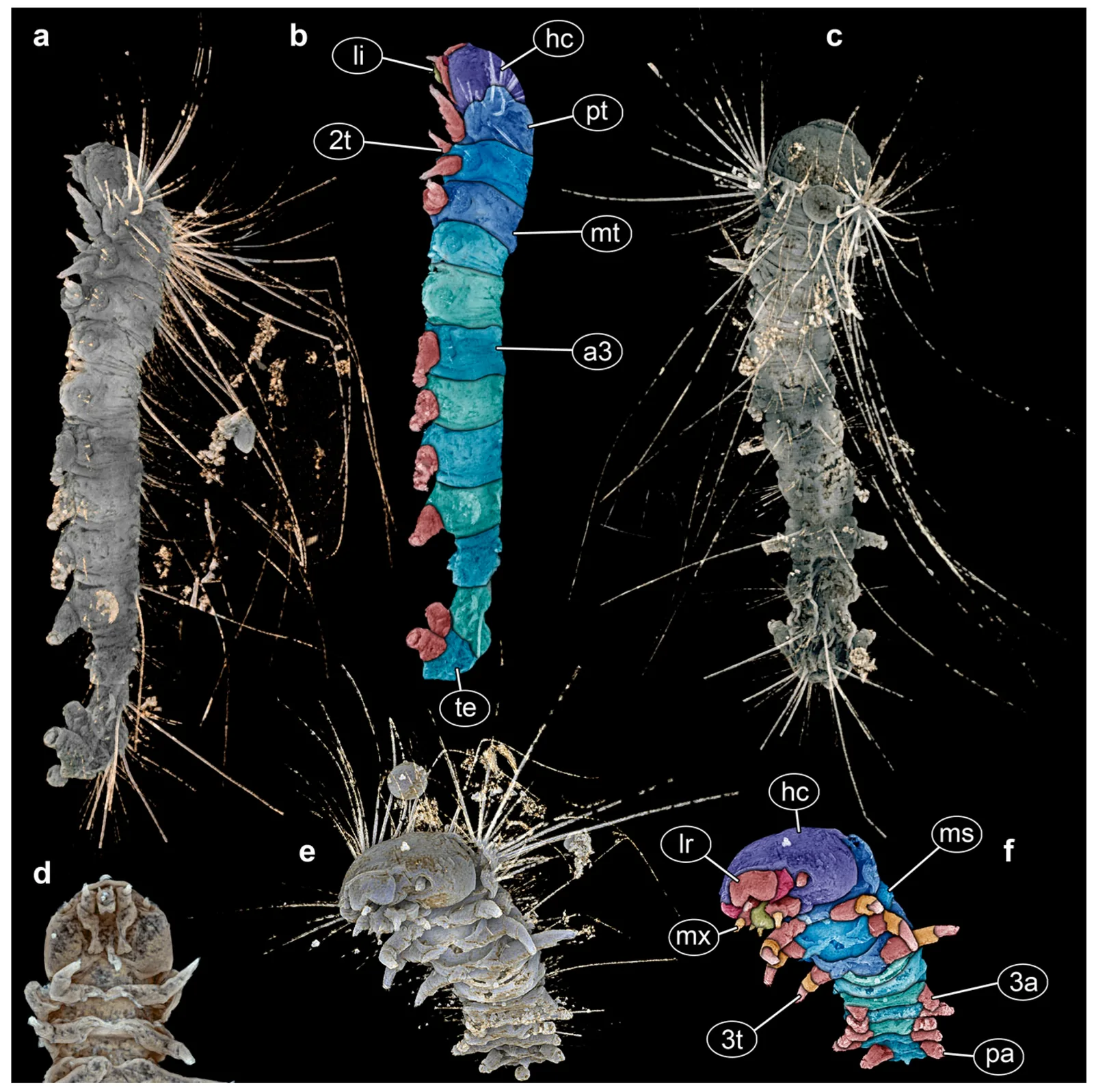
New caterpillar fossil, PED 1524, from Dominican amber, volume renderings from µCT scans. (**a**) Lateral view. (**b**) Color-marked version of (**a**). (**c**) Dorsal view. (**d**) Ventral view of anterior body region. (**e**) Antero-ventral view. (**f**) Color-marked version of (**e**). Abbreviations: 2–3t, appendage of thorax segment 2–3; 3a, appendage of abdomen segment 3; a3, abdomen segment 3; hc, head capsule; li, labium; lr, labrum; ms, mesothorax; mt, metathorax; mx, maxilla; pa, posterior appendage; pt, prothorax; and te, trunk end.

**Figure 6:**
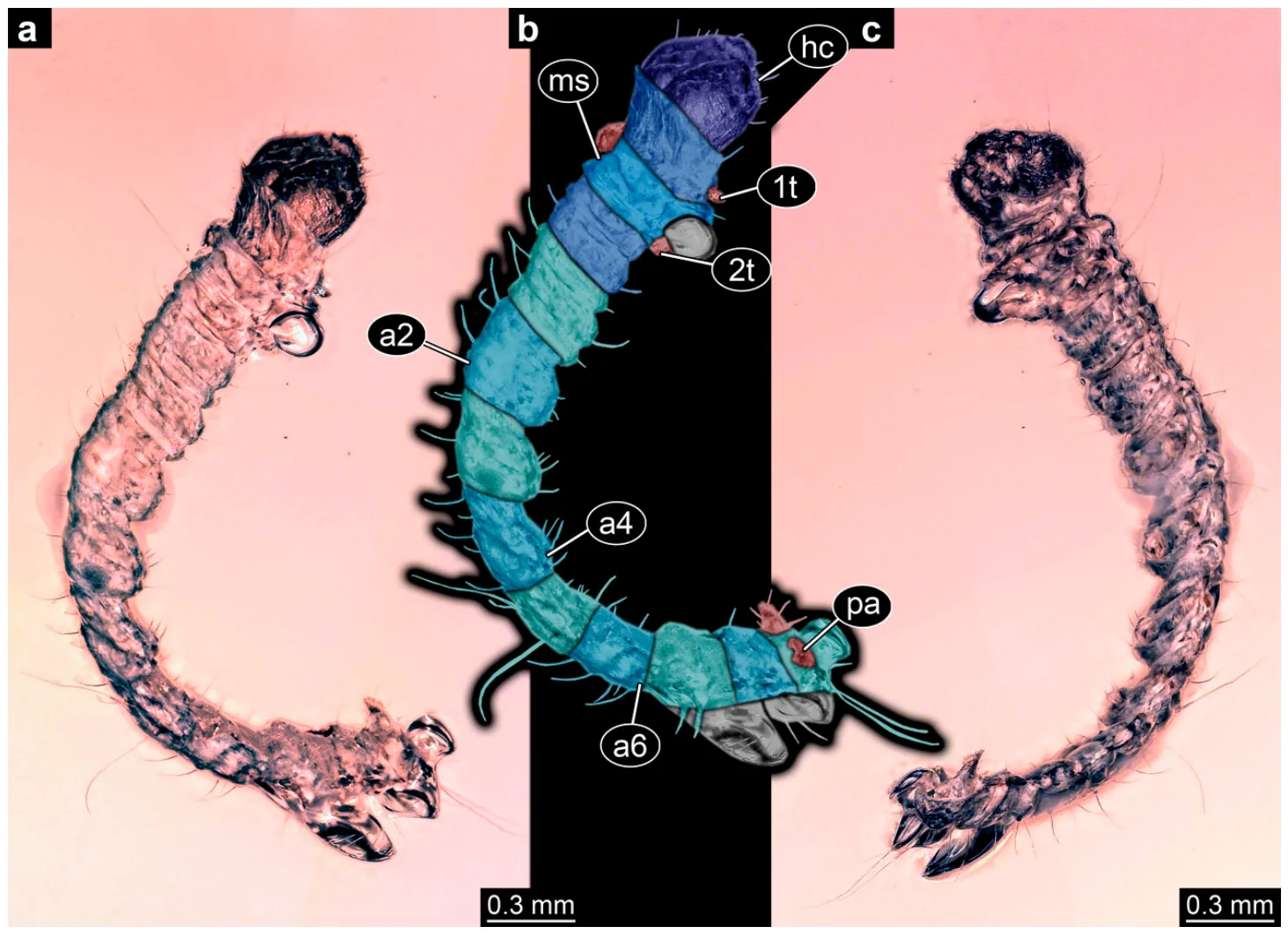
New caterpillar fossil, PED 1585, from Dominican amber. (**a**) Oblique dorsal view. (**b**) Color-marked version of (**a**). (**c**) Oblique ventral view. Abbreviations: 1–2t, appendage of thorax segment 1–2; a2–6, abdomen segment 2–6; hc, head capsule; ms, mesothorax; and pa, posterior appendage.

**Figure 7:**
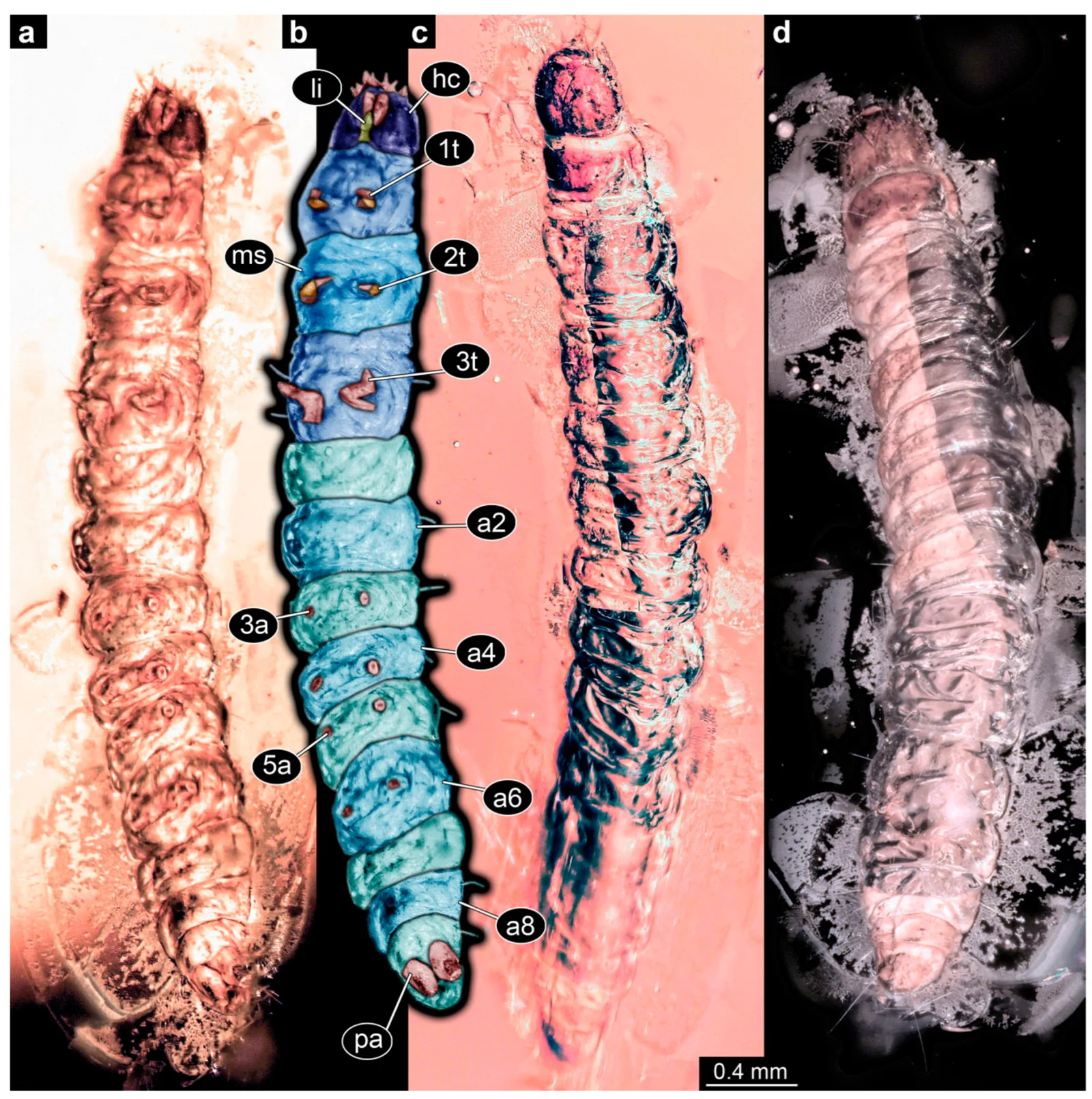
New caterpillar fossil, SMNS Do 359-K, from Dominican amber. (**a**) Ventral view. (**b**) Color-marked version of (**a**). (**c**) Dorsal view; note that the posterior end is much deeper in the amber. (**d**) Dorsal view, levelled horizontally with the help of glycerol. Abbreviations: 1–3t, appendage of thorax segment 1–3; 3–5a, appendage of abdomen segment 3–5; a2–8, abdomen segment 2–8; hc, head capsule; li, labium; ms, mesothorax; and pa, posterior appendage.

**Figure 8:**
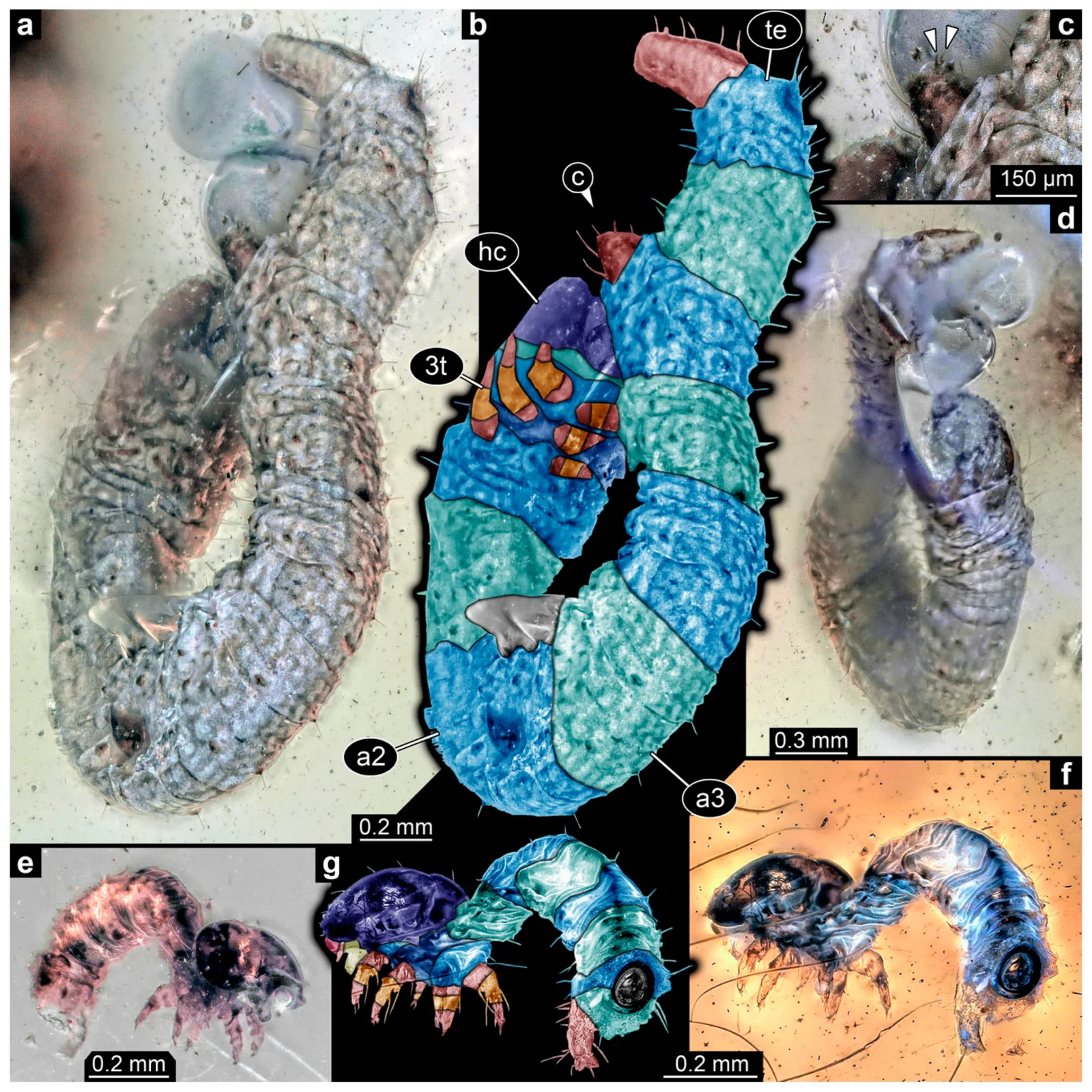
New caterpillar fossils, PED 2015, from Dominican amber, Geometridae. (**a**–**d**) PED 2015a. (**a**) Oblique ventral view. (**b**) Color-marked version of (**a**). (**c**) Detail of abdomen appendage 6 with few hooks (arrows). (**d**) Oblique dorsal view. (**e**–**g**) PED 2015b. (**e**) Antero-lateral view. (**f**) Postero-lateral view. (**g**) Color-marked version of (**f**). Abbreviations: 3t, appendage of thorax segment 3; a2–3, abdomen segment 2–3; hc, head capsule; and te, trunk end.

**Figure 9:**
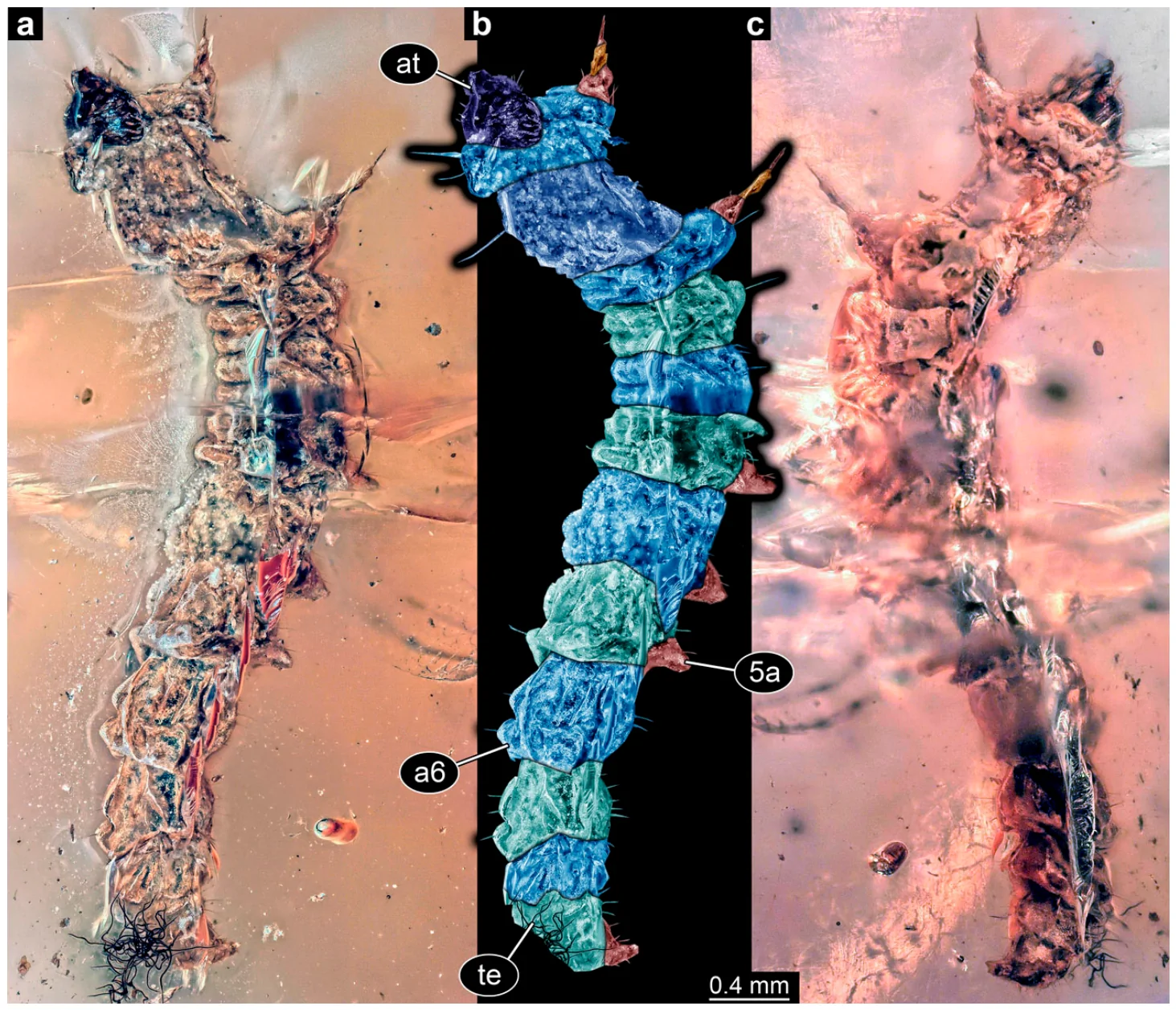
New caterpillar fossil, PED 2032, from Mexican amber. (**a**) Dorso-lateral view. (**b**) Color-marked version of (**a**). (**c**) Ventro-lateral view. Abbreviations: 5a, appendage of abdomen segment 5; a6, abdomen segment 6; at, antenna; and te, trunk end.

**Figure 10:**
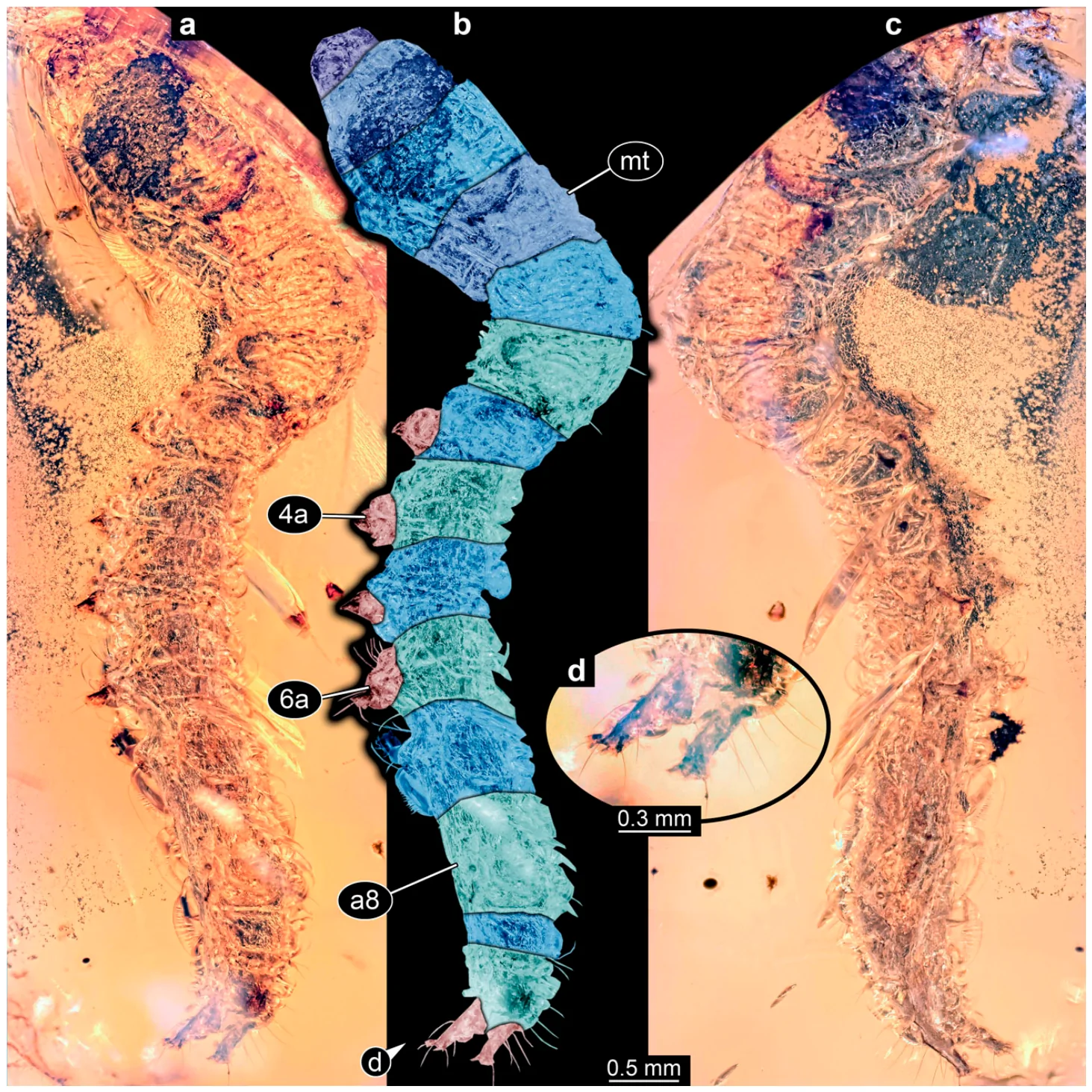
New caterpillar fossil, MEX 027, from Mexican amber. (**a**) Dorso-lateral view. (**b**) Color-marked version of (**a**). (**c**) Ventro-lateral view. (**d**) Close-up on terminal appendages. Abbreviations: 4–6a, appendages of abdomen segments 4–6; a8, abdomen segment 8; and mt, metathorax.

**Figure 11:**
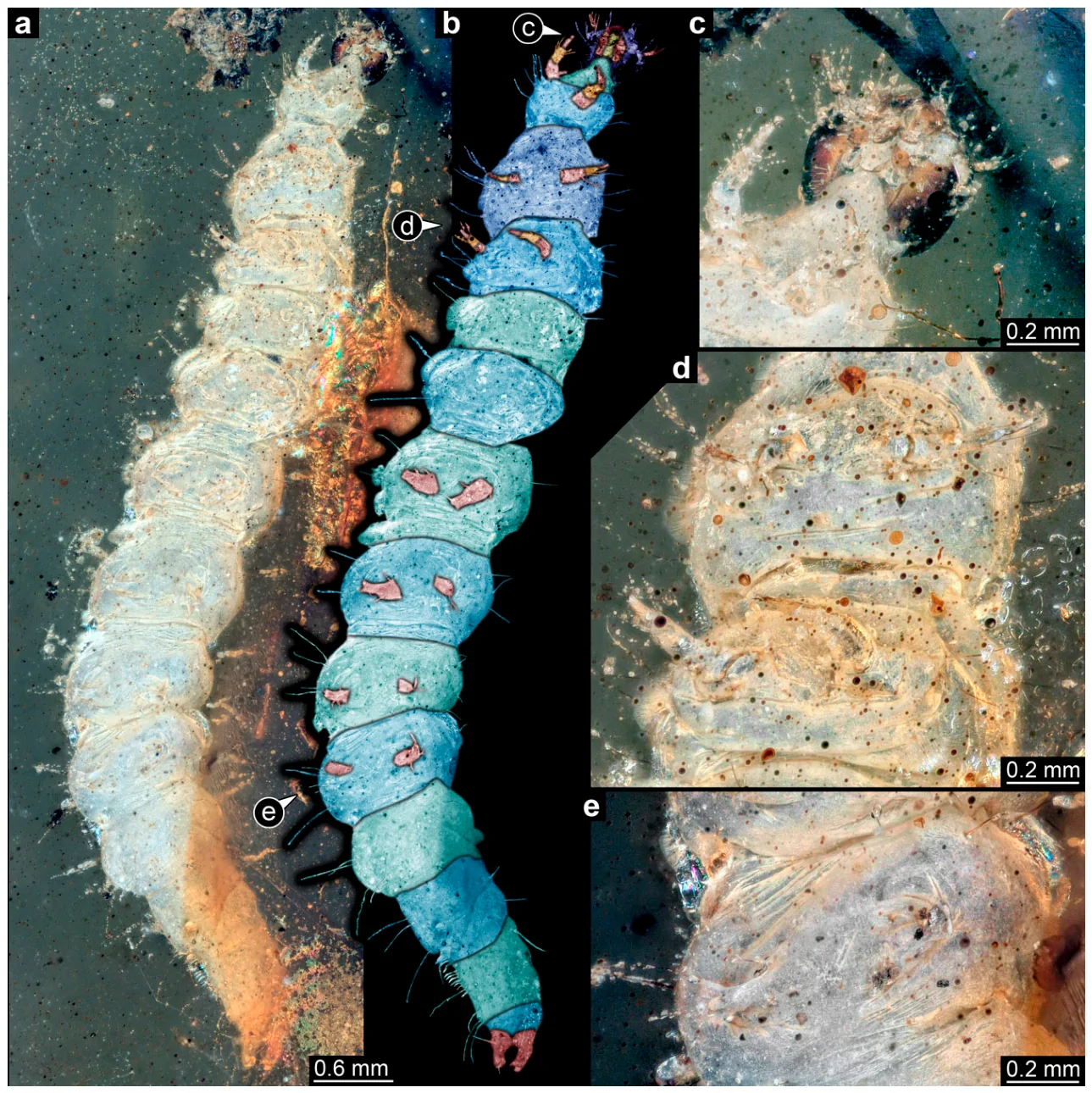
New caterpillar fossil, BUB 3968, from Kachin amber, Myanmar. (**a**) Ventral view. (**b**) Color-marked version of (**a**). (**c**) Detail of head. (**d**) Detail of thorax appendages. (**e**) Detail of abdomen appendages.

**Figure 12:**
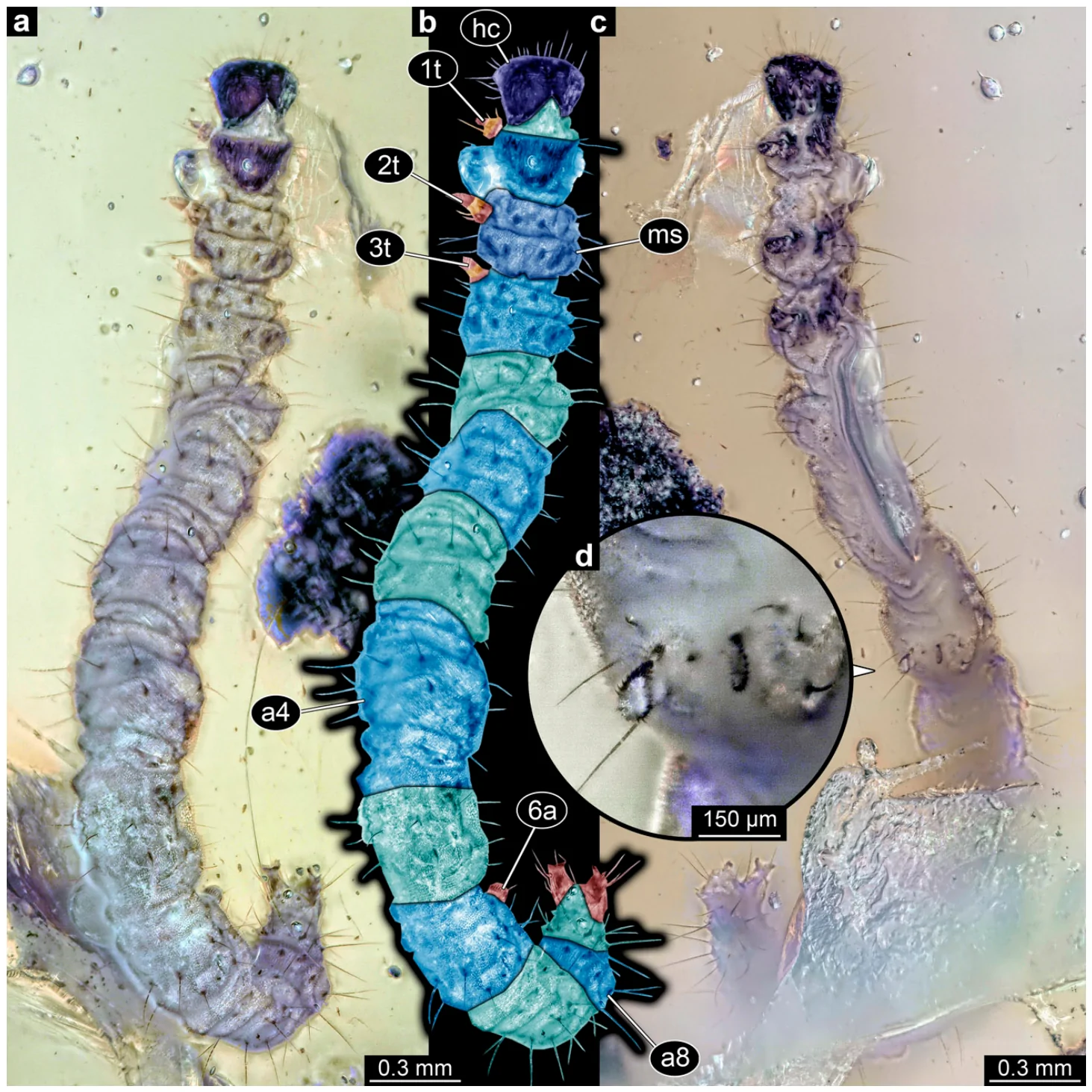
New fossil caterpillar, PED 2352, from Kachin amber, Myanmar. (**a**) Dorsal view. (**b**) Colour-marked version of (**a**). (**c**) Ventral view. (**d**) Detail of appendage of abdomen segment 4. Abbreviations: 1–3t, appendage of thorax segment 1–3; 6a, appendage of abdomen segment 6; a4–8, abdomen segment 4–8; hc, head capsule; and ms, mesothorax.

**Figure 13:**
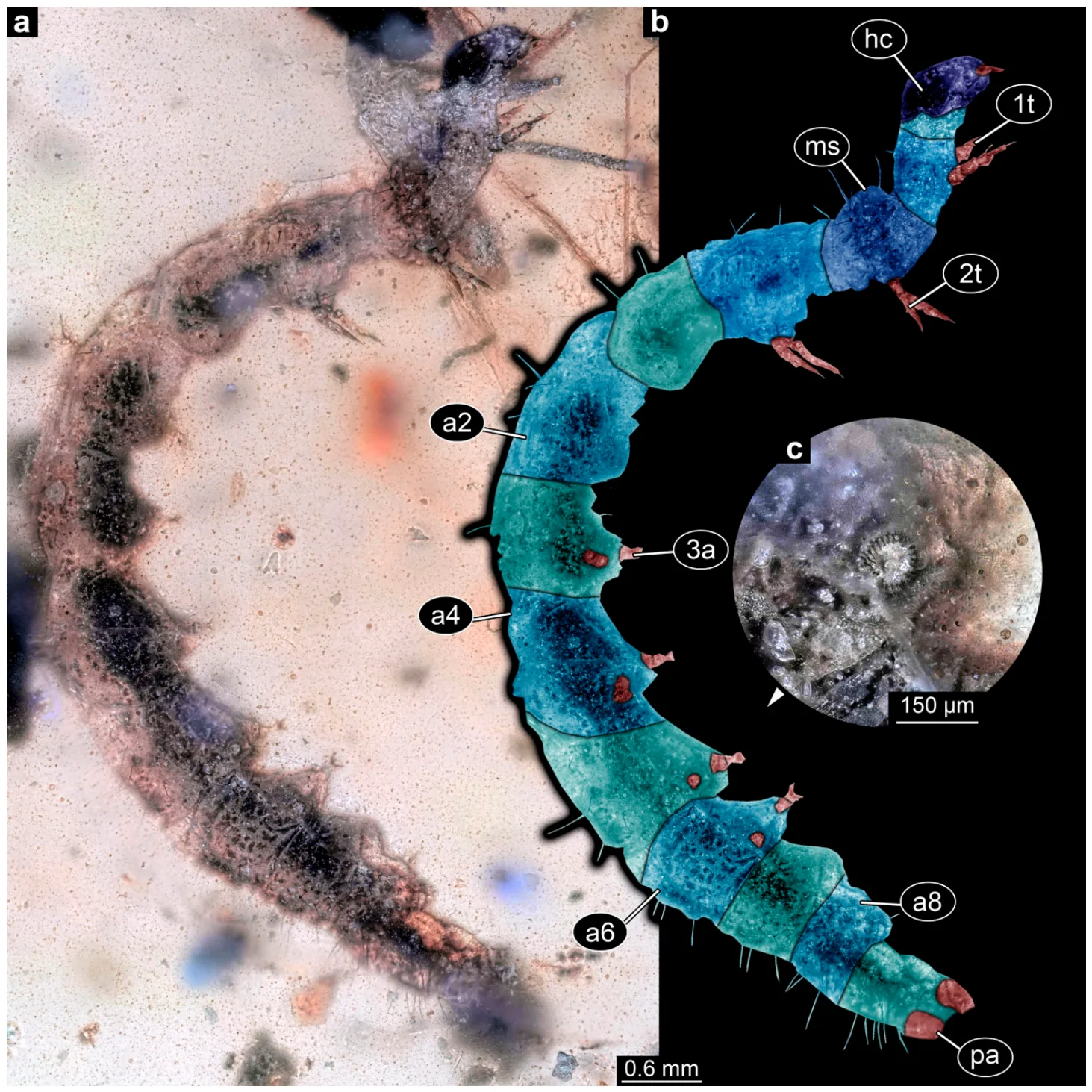
New caterpillar fossil, PED 4046, from Kachin amber, Myanmar. (**a**) Lateral view. (**b**) Color-marked version of (**a**). (**c**) Detail of appendage of abdomen segment 5. Abbreviations: 1–2t, appendage of thorax segment 1–2; 3a, appendage of abdomen segment 3; a2–8, abdomen segment 2–8; hc, head capsule; ms, mesothorax; and pa, posterior appendage.

**Figure 14:**
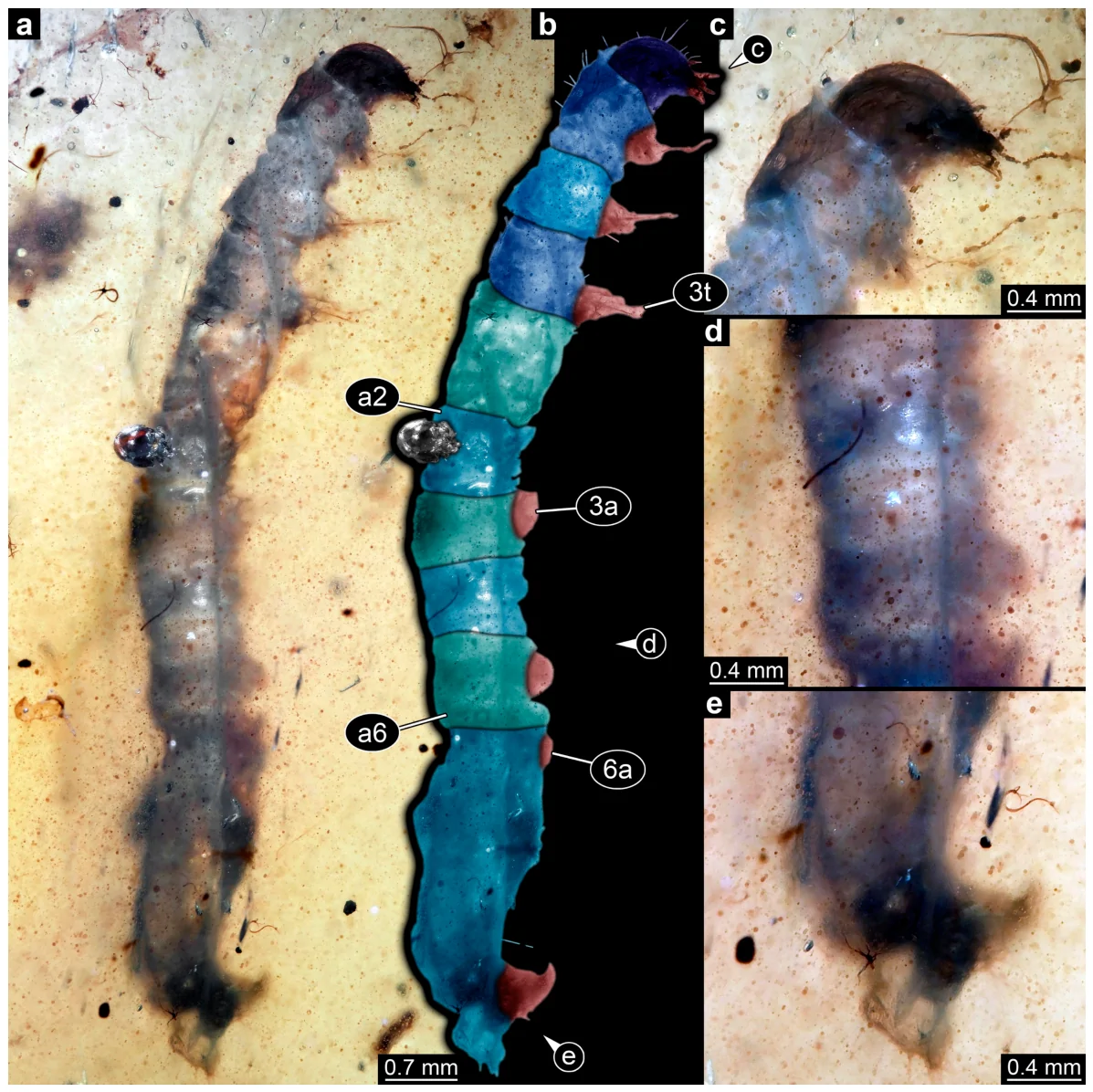
New caterpillar fossil, cn-05-25, from Kachin amber, Myanmar. (**a**) Lateral view. (**b**) Color-marked version of (**a**). (**c**) Detail of head. (**d**) Detail of prolegs. (**e**) Detail of posterior end with prolegs. Abbreviations: 3t, appendage of thorax segment 3; 3–6a, appendages of abdomen segments 3–6; and a2–6, abdomen segment 2–6.

**Figure 15:**
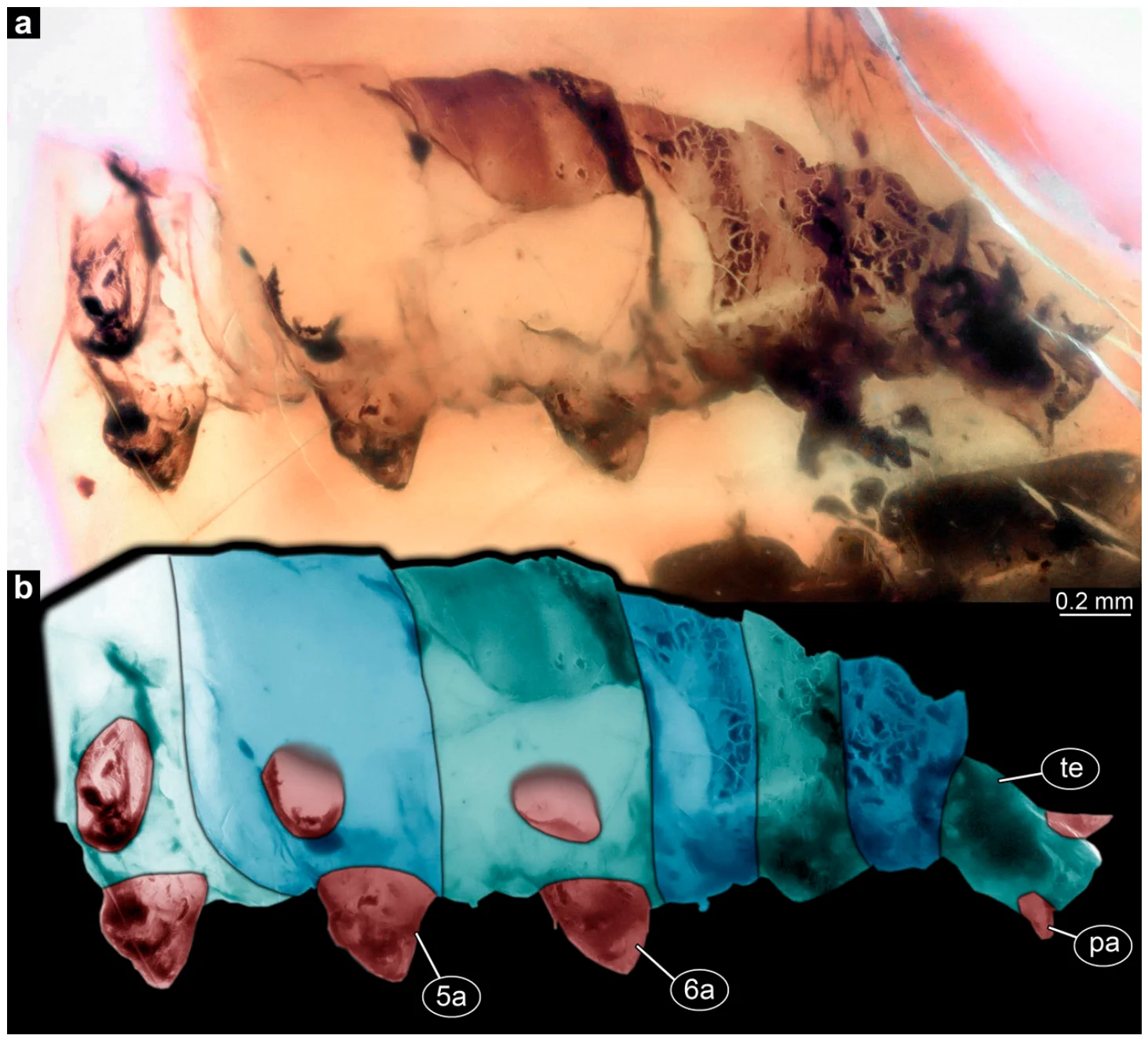
New caterpillar fossil, SMNS LB-250-2, from Lebanon amber. (**a**) Ventro-lateral view. (**b**) Color-marked version of (**a**). Abbreviations: 5–6a, appendage of abdomen segment 5–6; pa, posterior appendage; and te, trunk end.

**Figure 16:**
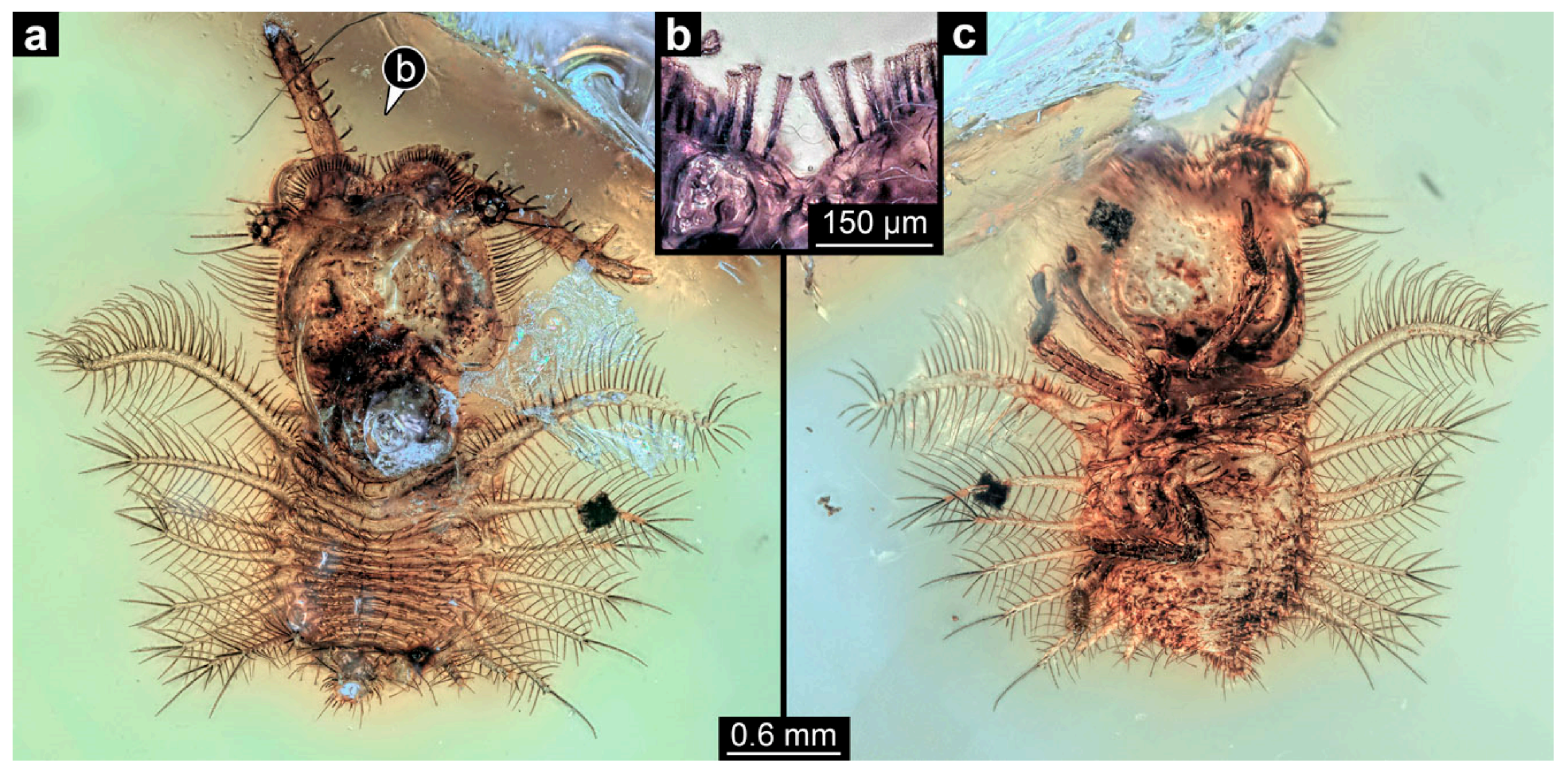
Comparative lacewing larva, CCGG 10059, from Dominican amber. (**a**) Overview in dorsal view. (**b**) Close-up on the anterior rim of the head. (**c**) Overview in ventral view.

**Figure 17:**
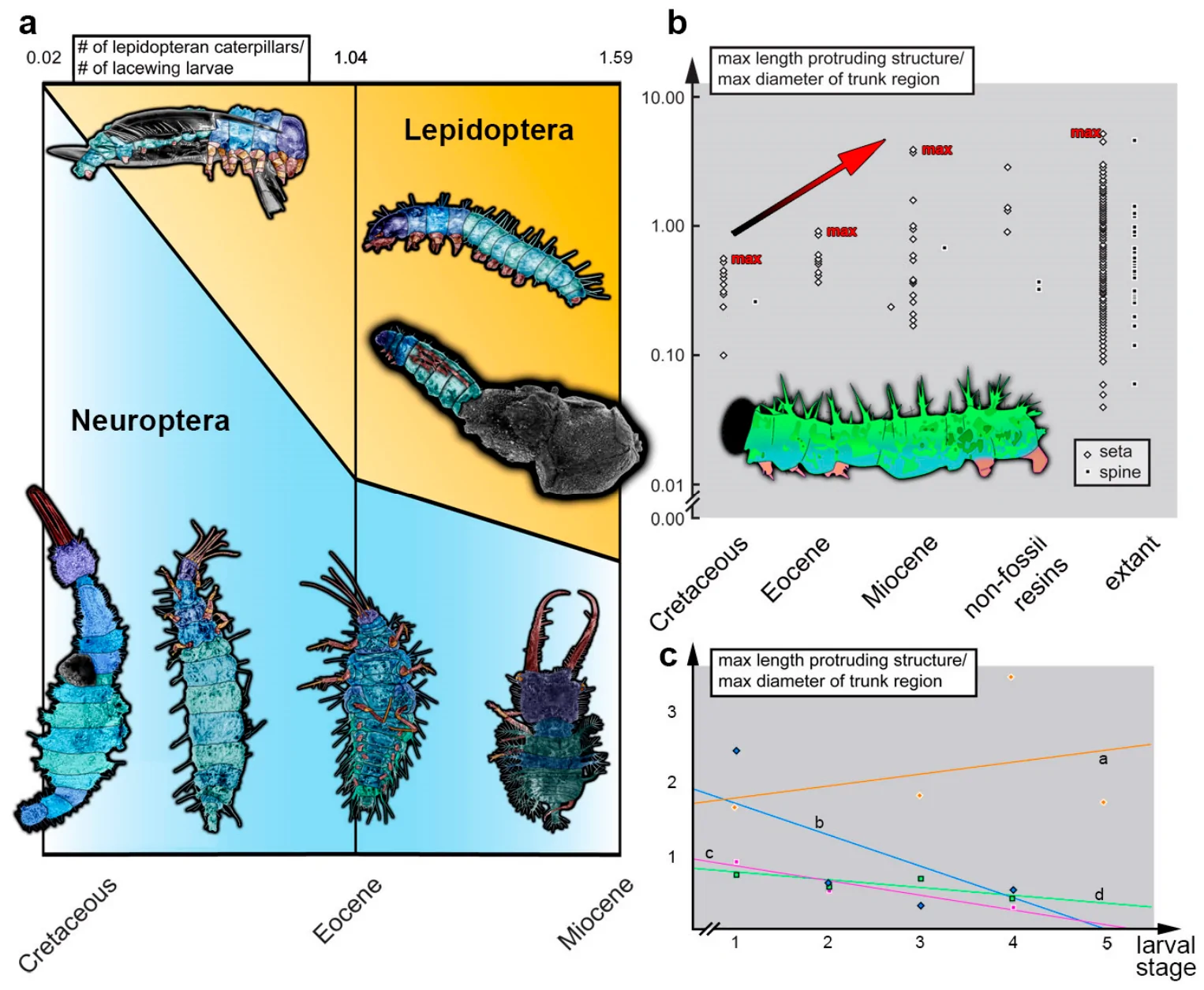
Quantitative aspects of caterpillars through time. (**a**) Relative abundances in comparison to lacewing larvae based on specimen numbers; ratios of caterpillar/lacewing larvae noted on top. (**b**) Relative lengths of protruding structures (setae, spines) in extant and caterpillar fossils of different geological ages. (**c**) Relative length of setae over larval stages for some extant species: *Casama innotata* (letter a) [110], *Duponchelia fovealis* (letter b) [111], *Hermeuptychia atalanta* (letter c) [112], and *Drepana arcuata* (letter d) [113]. Images re-used from [48,81,85,109,114].

## Data Availability

All data used in this study are included in the manuscript.

## Supplementary Materials

The following supporting information can be downloaded at https://www.mdpi.com/article/10.3390/insects17100991/s1, Supplementary Table S1: Sources for ratios of the measured setae and spines in relation to body diameter. Figure 1, Figure 2, Figure 3, Figure 4, Figure 5, Figure 6, Figure 7, Figure 8, Figure 9, Figure 10, Figure 11, Figure 12, Figure 13, Figure 14, Figure 15, Figure 16 and Figure 17 in high resolution are available at https://doi.org/10.5281/zenodo.19700093 (accessed on 22 April 2026).
